# Cognitive Impairments and Risk Factors After Ruptured Anterior Communicating Artery Aneurysm Treatment in Low-Grade Patients Without Severe Complications: A Multicenter Retrospective Study

**DOI:** 10.3389/fneur.2021.613785

**Published:** 2021-02-12

**Authors:** Ning Ma, Xin Feng, Zhongxue Wu, Daming Wang, Aihua Liu

**Affiliations:** ^1^Beijing Neurosurgical Institute, Beijing Tiantan Hospital, Capital Medical University, Beijing, China; ^2^Department of Neurosurgery, The First Hospital of Shanxi Medical University, Shanxi Medical University, Shanxi, China; ^3^Department of Neurosurgery, Beijing Hospital, Graduate School of Peking Union Medical College, Beijing, China

**Keywords:** intracranial aneurysms, microsurgical clipping, endovascular coiling, cognitive impairment, quality of life

## Abstract

**Background:** Cognitive impairment is the main factor affecting quality of life in patients with low-grade aneurysmal subarachnoid hemorrhage.

**Objective:** We explored cognitive impairments and risk factors after treatment for ruptured anterior communicating artery (AComA) aneurysms in low-grade (Hunt-Hess grade of 1–3) patients without severe complications.

**Methods:** One-hundred-twenty-six patients with a Hunt-Hess grade of 1–3 who underwent microsurgical clipping or endovascular embolization for ruptured AComA aneurysm treatment at three academic institutions in China from January 2015 to December 2017 were assessed with the modified Telephone Interview for Cognitive Status (TICS-m), the modified Rankin Scale (mRS), and the instrumental activities of daily living (IADL) scale 2 or more years after microsurgical clipping or endovascular coiling. Multiple cox-regression analysis was used to identify variables independently associated with cognitive impairment.

**Results:** Of the total of 126 patients, 115 (91.3%) achieved good clinical outcomes (mRS score 0–2) and 109 (86.5%) had excellent quality of life (IADL score 8). Twenty-eight (22.2%) patients showed cognitive impairments (TICS-m≤27). The multivariate COX regression analysis showed that the female patients and longer duration of loss of consciousness at onset of subarachnoid hemorrhage (SAH) were independently associated with cognitive impairment. Cognitive outcome at the latest follow-up was not significantly different between patients treated after surgical clipping and coiling.

**Conclusion:** About one in five patients showed cognitive impairments after treatment for ruptured AComA aneurysms. Patients who are of the female sex, and who have a longer duration of a loss of consciousness at the onset of SAH may be at risk of cognitive impairment.

## Introduction

SAH is a devastating subtype of stroke affecting relatively young people who have a mean premorbid life expectancy of 30 years. Case fatality can reach 35%, and many survivors experience long-term disability and cognitive impairments across multiple cognitive domains (1). Cognitive impairment may be a result of the transient cessation of circulation and the damage caused by blood entering the brain at the time of the aneurysm rupture (2, 3). The perfusion and metabolism in certain brain regions might be decreased by these factors, therefore the subsequent cognitive impairments could be attributed to the location of the aneurysm (4). Anterior communicating artery (AComA) aneurysms are the most common intracranial aneurysms and the most common sources of aneurysmal subarachnoid hemorrhage (aSAH) (5, 6). The AComA has several arterial branches that perfuse the basal forebrain, frontal lobe, anterior cingulate gyrus, septal nuclei, fornix, genu of the corpus callosum, and other neuroanatomical structures. Damage in both recent and remote memory, amnesia, confabulation, and personality changes were recorded as the main symptoms in the rupture of AComA aneurysms in earlier studies (4, 7). These symptoms can be caused by the intraoperative challenges unique to AComA-related aneurysms and by damaged anterior cerebral structures such as the frontal cortex, the ventromedial prefrontal (orbitofrontal) cortex, or the striatum (8).

Advances in care have decreased mortality, and survival rates after aneurysm rupture are reported at 65% and according to the Glasgow Outcome Scale(GOS), ≤80% of survivors are considered to have “good recovery” (9). Nevertheless, 46% of patients who suffer from aSAH may experience cognitive dysfunction, and 62% of patients with a GOS score of 1 may suffer cognitive impairment (10). More sophisticated tools reveal that ≤50% have neurocognitive deficits, usually in the absence of structural lesions (2, 11, 12). The combination of a younger age and high morbidity bring a heavy economic burden to both individuals and society. Furthermore, an estimated ≤50% of survivors will not return to the same level of work (13, 14).

Studies focusing on the long-term functional outcomes and quality of life after microsurgical clipping or endovascular embolization of ruptured AComA aneurysms in this young and productive population are rare.

The aim of our multicenter retrospective study is to evaluate the long-term cognitive outcomes of patients with low-grade aSAH and without severe complication after treatment for ruptured AComA aneurysms, and to analyze various associated factors using the modified Telephone Interview of Cognitive Status (TICS-m), the modified Rankin Scale (mRS), and the instrumental activities of daily living (IADL) scale. We also aim to compare cognitive outcomes in two groups of patients who underwent either microsurgical clipping or endovascular coiling.

## Methods

This multicenter retrospective follow-up study was conducted at three stroke institutes in China. Patients with ruptured AComA aneurysms who were admitted to these three institutions between January 2015 and December 2017 were reviewed. The inclusion criteria were as follows: (1) SAH caused by rupture of AComA aneurysm, confirmed by CT scanning and then by CT angiography or digital subtraction angiography and the mRS scores 0~2 in all patients before the aSAH; (2) early treatment (within 72 h) of ruptured AComA aneurysm by clipping or coiling; (3) no serious post-procedural complications (e.g., post-operative coma, hematoma, cerebral infarction, and hydrocephalus), and functional recovery allowing the patient to participate in the comprehensive neuropsychological battery of tests; (4) a Hunt and Hess grade of 1~3 at admission; (5) at least a primary school education level; and (6) post-operative follow-up for more than 2 years. The exclusion criteria were as follows: (1) a Hunt and Hess grade of 4~5; (2) a history of neurological or psychiatric disease; (3) cognitive impairments prior to the SAH; (4) below primary school education level or being illiterate; and (5) serious complications after the procedure and an inability to cooperate in cognitive assessments. All patients provided their consent to be included in the assessments and analyses after having been informed about the purpose of the study.

Demographic data included gender, age, smoking behavior, and education level. The initial neurological status and severity of aSAH were assessed using the Hunt and Hess scale. Vascular risk factors such as hypertension, diabetes, and hypercholesterolemia were defined based on the patient's history and/or use of relevant medication, and smoking was defined as whether the patient was smoking at the time of study entry. Aneurysm projection (superior/inferior) was determined by the relationship with the parent arteries on the lateral angiographical view. Superior type aneurysms are subdivided into anterior type (0–5 O'clock) and dorsal type (9–12 O'clock), and inferior type aneurysms (5–9 O'clock) (15). The anatomy of the anterior cerebral artery is divided into the following three types according to supply artery and according to the development of bilateral A1: double A1, left side A1, and right side A1.

### Management of SAH and Aneurysm Exclusion

All patients received conventional medical treatment for SAH, including calcium antagonists and daily transcranial Doppler monitoring, fluid and blood pressure management, pain treatment, necessary anticonvulsants, post-operative hypervolemia, and prevention of deep vein thrombosis (16).

Surgical or neuroradiologic treatment for aneurysms was performed as soon as possible, within 72 h after aneurysm rupture. The choice of surgical method between microsurgical clipping and endovascular coiling was discussed by both the interventional radiologist and the neurosurgeon. The optimal method of aneurysm treatment, decided on a patient-to-patient basis, was then proposed to the patient and relatives after discussion. Generally, right-sided approaches were chosen when the condition were appropriate however, a left-sided approach was used when the aneurysmal neck and the feeding arteries were more easily accessible from the left. Partial resection of the gyrus rectus was performed in patients with high-positioned aneurysms or in patients with limited surgical view and obvious brain swelling.

### Long-Term Follow-Up

Patients and their family members' telephone interviews were used in the follow-up assessment. Cognitive function was assessed using the TICS-m which is a 12-item questionnaire that provides a measurement of global cognitive function on the basis of verbal communication through telephone; scores range from 0 to 50, with higher scores indicating better function (17, 18). A score of 27 or lower is indicative of cognitive impairment (TICS-m≤27). Quality of life was assessed with the IADL questionnaire. The tool evaluates eight domains of daily capability: using a telephone using, using a means of transportation, shopping, managing medicine, handling finances, doing the laundry, preparing meals, and tidying the house. The scores of every item range from 0 to 1 based on the patient's ability in performing the corresponding activity. The final score is the sum of the eight items and ranges from 0 to 8, with 0 representing the lowest degree of autonomy and 8 the highest one.

### Statistical Analysis

Numeric variables were analyzed with independent-samples *t-*tests or the Mann-Whitney U-test. The difference between two means was calculated using the least squares method. Categorical variables were analyzed with chi-square tests or Fisher's exact test. All variables were included in the multiple Cox regression with a backward variable selection algorithm to identify the independent predictors of cognitive impairment at long-term follow-up. We determined the level of association between the predictive variables and cognitive impairment by the hazard ratio and the respective 95% confidence interval (HR, CI 95%). All statistical tests were two-tailed. A *P* < 0.05 was of statistical significance (unless otherwise indicated). Statistical analyses were performed with the SPSS 25.0 software package (SPSS Inc, Chicago, IL).

## Results

### Population

Between January 2015 and December 2017, 203 patients with ruptured AComA aneurysms of a Hunt and Hess grade between 1 and 3 were treated at the three included institutes. Of these, a total of 126 patients met the inclusion criteria and completed more than 2 years post-procedural follow-up. The mean age was 49.2 ± 9.9 years (ranging from 22 to 59 years), and 39.7% of the patients were female. Twenty seven patients (21.4%) underwent endovascular treatment, and 99 patients (78.6%) underwent microsurgical treatment. All enrolled patients underwent the cognitive assessment. The mean duration between procedure and assessment was 38.7 ± 9.4 months (ranging from 22 to 58 months).

### Functional Outcome and IADL

A total of 115 (91.3%) patients achieved good clinical outcomes (mRS scores 0–2). The distribution of patients into the distinct categories of the mRS was significantly different between the clipping and coiling groups (*P* = 0.042). 93.9% of patients in the microsurgical clipping group and 81.5% in the endovascular coiling group had mRS scores of 0–2. Regarding the evaluation of IADL, a total of 109 (86.5%) patients had excellent quality of life (IADL = 8); the mean IADL index score of all patients was 7.6 ± 1.2, and mean scores did not significantly differ between the microsurgical clipping and endovascular coiling groups (7.7 ± 0.9 vs. 7.2 ± 1.9, *P* = 0.179) (Table 1).

**Table 1:** Functional and cognitive outcome of patients after ruptured treatment of AComA aneurysms.

**mRS score on follow-up**	***N* (%)**
0	12 (9.5)
1	88 (69.8)
2	15 (11.9)
3	10 (7.9)
4	1 (0.8)
TICSm	
>27	98 (77.8)
≤27	28 (22.2)
IADL	
3	3 (2.4)
4	3 (2.4)
5	2 (1.6)
6	2 (1.6)
7	7 (5.6)
8	109 (86.5)

*mRS, the modified Rankin Scale*.

*TICSm, Telephone Interview for Cognitive Status*.

*IADL, instrumental activities of daily living*.

### Univariate Analysis and Multivariate Analyses for Risk Factors of Cognitive Outcome

Twenty-eight (22.2%) patients were found to have cognitive impairment, with TICS-m≤27, including seven (25.9%) in the coiling group and 21 (21.2%) in the clipping group. A univariate analysis was carried out to assess the association between cognitive outcomes and clinical and aSAH factors. Loss of consciousness, duration of loss of consciousness, Hunt-Hess grade, and education level were found to have a significant correlation with cognitive impairment (*P* < 0.05), and 28.6% of patients with gyrus rectus had cognitive impairments, though their association was non-significant. However, age, gender, hypertension, diabetes mellitus, dyslipidemia, heart comorbidities, smoking behavior, history of drinking, presence of hemorrhage, aneurysm size, treatment modality (microsurgical clipping vs. endovascular coiling), left-side approach, anatomy of the anterior cerebral artery, aneurysm projection, and mRS score on admission were not significantly associated with cognitive impairment (*P* > 0.05, Table 2).

**Table 2:** Univariate analyze showing the variables associated with cognitive impairment.

**Characteristic**	**TICS-m>27 n (%)**	**TICS-m≤27 n (%)**	***P*-value**
Female	35 (35.7)	15 (53.6)	0.089
Age≥ 50 (years)	44 (44.9)	17 (60.7)	0.140
Education			0.033
Elementary school	23 (23.5)	13 (46.4)	
Middle/High school	60 (61.2)	14 (50.0)	
University or more	15 (15.3)	1 (3.6)	
Hypertension	41 (41.8)	14 (50.0)	0.442
Diabetes mellitus	2 (2.0)	2 (7.1)	0.174
Dyslipidemia	3 (3.1)	1 (3.6)	1.000
Drink	32 (32.7)	9 (32.1)	0.933
Heart comorbidities	27 (27.9)	8 (28.6)	0.776
Smoking	38 (38.8)	10 (35.7)	0.740
History of drinking	17 (17.8)	4 (14.3)	0.453
Hunt and Hess Grade			0.042
1~2	82 (83.7)	19 (77.9)	
3	16 (16.3)	9 (32.1)	
Loss of consciousness			0.001
Absent	73 (74.5)	11 (39.3)	
Present	25 (25.5)	17 (60.7)	
Duration of Loss of consciousness			0.046
<10 min	10 (40.0)	2 (11.8)	
10~60 min	9 (36.0)	5 (29.4)	
>60 min	6 (24.0)	10 (58.8)	
Presence of hemorrhage			0.153
Cisternal SAH	55 (56.1)	12 (43.9)	
Parenchymal hematoma and/or IVH	43 (42.9)	16 (57.1)	
Treatment modality			0.602
Endovascular coiling	20 (20.4)	7 (25.0)	
Microsurgical clipping	78 (79.6)	21 (75.0)	
Left-side microsurgical clipping	42 (42.9)	16 (57.1)	0.181
Stent assisted coiling	10 (10.2)	1 (3.6)	0.268
Gyrus rectus	17 (17.3)	8 (28.6)	0.098
mRS score on admission			0.189
0–2	81 (82.7)	20 (71.4)	
3–5	17 (17.3)	8 (28.6)	
Aneurysm size			0.126
<5 mm	49 (50.0%)	20 (71.4)	
5–10 mm	40 (40.8)	7 (25.0)	
>10 mm	9 (9.2)	1 (3.6)	
Anatomy of the anterior cerebral artery			0.28
Double A1	26 (26.5)	8 (28.6)	
Left side A1	48 (49.0)	17 (60.7)	
Right side A1	24 (24.5)	3 (10.7)	
Aneurysm projection			0.498
Inferior type	42 (42.9)	10 (35.7)	
Superior type	56 (57.1)	18 (64.3)	

*TICSm, Telephone Interview for Cognitive Status*.

*SAH, Subarachnoid Hemorrhage*.

*IVH, Intraventricular Hemorrhage*.

According to the multivariate COX regression analysis, female (HR: 2.7; 95% CI: 1.2–6.0; *P* = 0.013) and longer duration of loss of consciousness (HR: 7.9; 95% CI: 3.3–19.0; *P* = 0.000) were independently associated with cognitive impairment (Table 3).

**Table 3:** Multivariate cox regression model showing the independent predictive variables of cognitive impairment in 126 low-grade patients after ruptured AComA aneurysms treatment.

**Variable**	**Hazard ratio (95%CI)**	***P-*value**
Female	2.7 (1.2–6.0)	0.013
Duration of Loss of consciousness		
Absent		0.000
<10 min	0.8 (0.2–3.9)	0.787
10~60 min	2.8 (0.9–8.1)	0.065
>60 min	7.9 (3.3–19.0)	0.000

### Comparison of Quality of Life and Cognitive Outcome Between Microsurgical Clipping and Endovascular Coiling Group

The baseline clinical characteristics, clinical outcomes, and questionnaire outcomes were stratified based on treatment modality in the questionnaire completed by the patients (Table 4). There was no significant difference between patients treated with microsurgical clipping and endovascular coiling in demographics and baseline clinical characteristics (Table 4). Additionally, patients treated with microsurgical clipping had higher mRS scores (more than 2) than patients treated with endovascular coiling (6.1 vs. 18.5%, respectively, *P* = 0.042). However, those treated with microsurgical clipping had similar rates of TICS-m scores at the latest follow-up than patients treated with endovascular coiling (mean TICS-m score: 33.0 ± 6.4 vs. 33.9 ± 8.4, *P* = 0.912; TICS-m≤27: 21.2 vs. 25.9%, *P* = 0.602). Moreover, the mean IADL score of the microsurgical group was lower than that of the endovascular group, but the difference was not statistically significant (7.7 ± 0.9 vs. 7.2 ± 1.9, respectively, *P* = 0.179).

**Table 4:** Comparison of clinical, radiological characteristics, functional, and cognitive outcome between open surgery and coiling modalities.

**Characteristic**	**Endovascular coiling n (%)**	**Microsurgical clipping n (%)**	***P*-value**
Total	27 (21.4)	99 (78.6)	
Female	14 (51.9)	36 (36.4)	0.145
Age> 50 (years)	15 (55.6)	46 (46.5)	0.402
Education			0.347
Elementary school	8 (29.6)	28 (28.3)	0.547
Middle/High school	14 (51.9)	60 (60.6)	
University or more	5 (18.5)	11 (11.1)	
Hypertension	15 (55.6)	40 (40.4)	0.159
Diabetes mellitus	1 (3.7)	3 (3.0)	0.860
Dyslipidemia	2 (7.4)	2 (2.0)	0.201
Heart comorbidities	26 (53.1)	0(0.0)	0.376
Smoking	8 (29.6)	40 (40.4)	0.307
Drink	5 (18.5)	36 (36.4)	0.079
History of drinking	3 (17.6)	17 (22.1)	0.453
History of SAH	25 (11.7)	6 (7.8)	0.337
Hunt and Hess Grade			
1–2	23 (85.2)	78 (78.8)	0.309
3	4 (14.8)	21 (21.2)	
Aneurysm size			0.569
≤ 5 mm	19 (70.4)	50 (50.5)	0.074
5–10 mm	5 (35.3)	42 (42.4)	
>10 mm	3 (11.1)	7 (7.1)	
mRS score 3–5 on admission	2 (7.4)	15 (15.2)	0.296
mRS >2 on follow-up	5 (18.5)	6 (6.1)	0.042
TICS-m 33.9 ± 8.4	33.0 ± 6.4	0.912	
≤ 27	7 (25.9)	21 (21.2)	0.602
IADL, mean	7.2 ± 1.9	7.7 ± 0.9	0.179
IADL <8	6 (22.2)	11 (11.1)	0.134

*mRS, the modified Rankin Scale*.

*TICSm, Telephone Interview for Cognitive Status*.

*IADL, instrumental activities of daily living*.

## Discussion

Cognitive impairments reduce a patient's quality of life, participation in work, and social activities. Many studies have compared cognitive outcomes which do not exclude patients with very poor-grade SAH or with serious complications such as hydrocephalus, delayed cerebral ischemia, and intracranial rebleeding (3, 11, 19–21). However, few studies have directly explored the functional outcomes of young patients with good-grade SAH. AComA aneurysms are among the most identified ruptured aneurysms (22, 23), and AComA aneurysm rupture and treatment are more strongly associated with cognitive and behavioral deficits than aneurysms at other locations (23). In this cohort of patients with ruptured AComA aneurysms, we therefore excluded patients with poor-grade SAH (Hunt and Hess grade 4–5), post-operative coma, hydrocephalus, delayed cerebral ischemia, and intracranial rebleeding to control for the possible confounding effects of these factors.

### Factors Associated With Quality of Life and Cognitive Outcome Between Microsurgical Clipping and Endovascular Coiling

The study of Shen et al. (24) investigated 152 patients with aneurysmal SAH which was treated by endovascular coiling and they found that 59 patients (39%) developed cognitive impairment 6 months later. The authors also reported that AComA aneurysms, delayed cerebral ischemia, and hydrocephalus were independently associated with a higher risk of mild cognitive impairment after aneurysmal SAH (24). After adjustments for age, Wong et al. (25) found that admission of World Federation of Neurosurgical Societies (WFNS) grade, mode of aneurysm treatment, and delayed cerebral infarction were independently associated with poor cognitive outcomes. After excluding patients with poor Hunt-Hess grades (4–5), post-operative hydrocephalus, intracranial rebleeding, and symptomatic delayed cerebral ischemia, the multivariate COX regression analysis showed that being female, and having a longer duration of loss of consciousness at onset of subarachnoid hemorrhage were independent risk factors associated with cognitive impairment after treatment for ruptured AComA aneurysms. In a univariate analysis, loss of consciousness and the duration of the coma caused by SAH after the AComA aneurysm ruptured in such patients, were significantly associated with the cognitive outcome. Moreover, in a multivariate analysis, we found that the longer duration of loss of consciousness was obviously associated with cognitive impairment. We often encounter studies on the relationship between traumatic brain injury with loss of consciousness and cognitive imparient (26), however there are only a few studies that investigate the relationship between loss of consciousness of aSAH and cognitive outcome. As we know, loss of conscious is the representative symptom of aSAH. The cerebral blood flow could be reduced due to vasospasm and cerebral edema and it can lead to a coma in the initial stage of the subarachnoid hemorrhage. This result deserves further extensive study. What should be noted in our study is that the gyrus rectus has been found to not be significantly associated with cognitive outcome. This result is the same as the conclusion reached in another relevant study (27). This finding needs to be verified by further prospective large data research. Interestingly, a higher education level was significantly associated with cognitive outcome in the univariate analysis, however, the association was non-significant in the final multivariate analysis. Although this result is consistent with a previous study (3), it may also be a result of the small sample size. More detailed and in-depth large-scale prospective studies need to further explore their relationship.

### Comparison of Quality of Life and Cognitive Outcome Between Microsurgical Clipping and Endovascular Coiling

For ruptured AComA aneurysms, endovascular coiling may initially be proposed when the configuration is suitable, but microsurgical clipping remains an option that allows the patient to attain the same quality of life, functional outcome, and executive function (28). Many studies suggest that microsurgical clipping may lead to more severe cognitive impairment and higher rates of patient dependency compared with endovascular coil embolization, possibly caused by retraction injury to the frontal lobe or other causes of cerebral infarction (29–31). Frontal lobe infarction and SAH infarction are more common after surgical clipping of ruptured AComA aneurysms, and coiled patients have better outcomes at discharge and are more likely to be functionally independent than clipped patients (32). However, results on the cognitive functions of young and low-grade patients remain limited, and studies analyzing cognitive outcomes according to treatment procedure are rare. Thus, our analyses provide a unique opportunity to compare the cognitive outcomes of coiling and clipping treatment for the AComA location. In this study with a long-term follow-up of more than 2 years, there was no difference in excellent outcomes (mRS scores 0–1) between patients with microsurgical clipping and patients with endovascular coiling. The mean IADL score was higher in the microsurgical group than in the endovascular group, but this difference was not statistically significant. As for cognitive outcomes, those treated with microsurgical clipping had similar TICS-m scores at the latest follow-up than patients treated with endovascular coiling. These results suggest that clinical outcomes (measured with the mRS and the IADL) and cognitive function outcomes between microsurgical clipping and endovascular treatment groups are similar.

### Limitations

There are several limitations to our study. First, although we excluded patients with symptomatic delayed cerebral ischemia, this study did not consider brain damage on post-operative MR imaging (because only a subset of patients had undergone MRI scanning). Thus, we were unable to assess whether patients had cerebral infarctions after treatment, which might affect cognitive outcomes. Moreover, we excluded a lot of patients at risk of cognitive impairment such as hydrocephalus. Thus, we may underestimate the prevalence of cognitive impairment in our patients with a good outcome. Secondly, the size of the patient group did not allow for subgroup analyses of age and aneurysms size. Thirdly, our study did not use other reported cognitive screening tests such as the Montreal Cognitive Assessment (33) or the Mini-Mental State Examination (34). This is because these screening tests require face-to-face interviews, which are difficult to conduct in a group of patients from all over the country. Fourth, only 27 patients (21.4%) underwent endovascular treatment in our three centers. Fifth, as patients did not complete cognitive evaluations of TICS-m when they were admitted to the hospital, we were unable to retrospectively determine whether they had cognitive impairments before onset or surgery. Finally, we did not achieve sequential follow-up at different periods that would have contributed toward a better understanding of recovery of cognitive functions.

## Conclusions

Using an easy-to-use cognitive assessment tool (TICS-m) by phone without face-to-face interactions, our results suggest that being of the female sex, and having a longer duration of loss of consciousness at onset of subarachnoid hemorrhage were independent risk factors associated with cognitive impairment after treatment for ruptured AComA aneurysms. No significant differences of cognitive outcome could be detected between the microsurgical clipping and endovascular coiling group.

## Data Availability Statement

The original contributions presented in the study are included in the article/supplementary material, further inquiries can be directed to the corresponding author/s.

## Ethics Statement

The studies involving human participants were reviewed and approved by ethics committee of Beijing Tiantan Hospital, Capital Medical University. The participants provided written informed consent to participate in this study.

## Author Contributions

Material preparation, data collection, and analysis were performed by NM, XF, and AL. The first draft of the manuscript was written by NM. All authors contributed to the study conception and design, commented on previous versions of the manuscript, and read and approved the final manuscript.

## Conflict of Interest

The authors declare that the research was conducted in the absence of any commercial or financial relationships that could be construed as a potential conflict of interest.
